# Austria; Its Literary, Scientific, and Medical Institutions, with Notes upon the Present State of Science, and Guide to the Hospitals and Sanatory Establishments of Vienna

**Published:** 1843-08-05

**Authors:** 


### BIBLIOGRAPHICAL NOTICES.

*Austria; its Literary, Scientific, and Medical Institutions, with Notes upon the Present State of Science, and Guide to the Hospitals and Sanatory Establishments of Vienna.* By W. R. Wilde, M. R. I. A., Licentiate of the Royal College of Surgeons, Ireland, &c., &c. Dublin, London, and Edinburgh: 1843. 8vo. pp. 325.

Mr. Wilde has written an instructive and entertaining book; one that was much wanted, and which no one better than himself could undertake and accomplish. His experience as a traveller, united to his powers of observation, and great readiness of mind, fit him peculiarly well for a work of this kind. The school of Vienna is fast rising into notice, and will, we venture to predict, in a few years, supplant that of Paris, as the latter has superseded that of Edinburgh. No where are the opportunities for clinical instruction approached to the degree that they may be enjoyed in Vienna; and the Professors are all men of high attainments and great industry. The want of a suitable Hand-Book is much felt in the Austrian capital by the stranger, for no good guides existed in any language before the present publication, and the difficulties of obtaining accurate information on any subject connected with the condition of the state cannot be understood except by those who have undergone the inconvenience. The introduction of foreign works is almost prohibited; all the native publications have the awe of the censorship before them, and if the traveller openly avows that the object of his visit is information, every obstacle will be thrown in his way.

The first part of Mr. Wilde's book is occupied with a description of the school system of Austria, which is, as a whole, so admirable. The founder of the present system of national education was the celebrated Van Swieten, the physician to the Empress Maria Theresa. Education is compulsory, and the laws against ignorance are so severe, and so strictly enforced, that few are disposed to subject themselves to their action. Religious instruction is an integral part of the system. The Roman Catholic is the established religion, but dissenters, as well as Jews, partake equally of the benefits of the schools, without being obliged to assist in the religious exercises, they arriving and departing an hour sooner or later.

That part of the book which treats of the School of Medicine is the one of which we propose here to give our readers some acount.

The medical faculty consists, besides the municipal officers, of sixteen ordinary and nine extraordinary professors, besides numerous adjuncts. The course is as follows:—

| | COURSE. | SUBJECT. | PROFESSOR. | HOUR. | FREQUENCY IN THE WEEK. |
|---|---|---|---|---|---|
| First Year. | First Semester. | Introduction to the Medical Studies and Mineralogy | Dr. C. Fischer. | 10—11 | 5 times. |
| | | Anatomy | Dr. Joseph Berres. | 9—10 | do. |
| | | Zoology | Dr. C. Fischer. | 10—11 | do. |
| | | Botany | Dr. S. Endlicher. | 7½—8½ | do. |
| | | Anatomy | Dr. Joseph Berres. | 9—10 | do. |
| | Second Semester. | Anatomical Dissections | | | |
| Second Year. | First Semester. | Anatomy and Physiology (in Latin) | Dr. J. Czermak. | 10—11 | do. |
| | | General Chemistry | Dr. Ad. Pleischl. | 11—1 | do. |
| | Second Semester. | Anatomy and Physiology | Dr. J. Czermak. | 10—11 | do. |
| | | Pharmacy | Dr. Ad. Pleischl. | 11—1 | do. |
| Third Year. | First Semester. | General Pathology and Etiology (in Latin) | Dr. S. Töltenyi. | 3—4 | Daily. |
| | | Materia Medica et Chirurgica, Therapea, Diatetic, and the Art of Prescribing (in Latin) | Dr. S. Töltenyi. | 8—9 & 3—4 | do. |
| | | Theory of Midwifery (in the Krankenhaus) | Dr. John Klein. | 12—1 | 5 times. |
| | Second Semester. | Contagious Diseases of Animals,—in Veterinerary Institute | Dr. Ant. Hayne. | 5¼—6¼ | 3 times. |
| Fourth Year. | First Semester. | Medical Clinique (in the Krankenhaus) | Dr. Hildenbrand. | 8—9 | Daily. |
| | | Special Therapea for Internal Diseases | do. | 9—10 | 5 times. |
| | | Surgical Clinique | Dr. Wattmann. | 10—11 | Daily. |
| | | Surgical Operations | do. | 11—12 | 5 times. |
| | Second Semester. | Medical Clinique | Dr. Hildenbrand. | 11—12 | do. |
| | | Therapea, as above | do. | 9—10 | do. |
| | | Surgical Clinique | Dr. Wattmann. | 10—11 | Daily. |
| | | Special Surgical Pathology | do. | 11—12 | 5 times. |
| Fifth Year. | First Semester. | Medical Clinique | Dr. Hildenbrand. | 8—9 | Daily. |
| | | Special Therapea for Internal Diseases | do. | 9—10 | 5 times. |
| | | Ophthalmic Clinique | Dr. Rosas. | 10—11 | Daily. |
| | | Ophthalmic Science | do. | 11—12 | 5 times. |
| | | Legal Medicine | Dr. Joseph Brent. | 12—1 | do. |
| | | Exercise in Judiciary Dissections | do. | | |
| | Second Semester. | Medical Clinique | Dr. Hildenbrand. | 8—9 | Daily. |
| | | Special Therapea for Internal Diseases | do. | 9—10 | 5 times. |
| | | Ophthalmic Clinique | Dr. Rosas. | 10—11 | Daily. |
| | | Ophthalmic Surgery | do. | 11—12 | 5 times. |
| | | Medical Police | Dr. Joseph Brent. | 7—8 | do. |
| | | Judiciary Dissections | do. | | |

The term of study is five years, and none are permitted to undertake it who have not first class certificates for the final examination of their philosophical studies.

"Students wishing to take out the degree of doctor of surgery are obliged, in addition to this course, to attend the surgical clinique, and the lectures upon the practice of surgery, during the fifth year: but as these lectures take place at the same hour as the ophthalmic clinique, those pupils are compelled to attend the latter in the next ensuing season, that is, the first semester of the sixth year. Those who wish to take out a special diploma, as *Augenarzt*, are obliged to repeat the second semester of the ophthalmic course; and those who wish to become master-accoucheurs are required to attend for two additional months in the obstetric clinique, and also to undergo an especial examination."

The course, as it will appear, is extensive, and well-arranged, and the student is obliged to adhere strictly to the prescribed routine. At the end of each semester, the students are examined by both the clinical and special professors, before they are permitted to pass to a higher grade. Anatomy does not command at Vienna the same attention as at Paris or Berlin. The celebrated Berres is the Professor. Physiology is very imperfectly studied, both the religion of the country, and the censorship frowning upon it.

The clinical school of Vienna has long been celebrated, and is at present the most renowned in Europe. It consists of four schools—the medical clinic for physicians; the medical clinic for surgeons; and the surgical and ophthalmic clinics. Mr. Wilde thus describes the medical clinic:—

"The medical clinique for physicians is held from eight to ten o'clock, on five days in the week, in a distinct building of the *Krankenhaus*, which faces the entrance into the first great quadrangle; it consists of a male and female ward, containing together twenty-eight beds, with an average reception of from two hundred and eighty to three hundred patients annually; these patients always consist of the most interesting and instructive cases selected from the general wards of the hospital by the professor's assistant, who, on a vacancy occurring in the clinique, has the privilege of removing into it any patient he chooses—each new case is immediately delivered into the hands of a pupil, taken in rotation, and styled the *Ordinarius*, who carefully notes the symptoms, and all the phenomena of disease then presenting, and who prescribes, under the superintendence of the assistant, any medicine that may be immediately necessary. On the next visit of the professor, the *Ordinarius* examines the patient in the presence of the class, inquiring minutely into the origin and history of the case—the state of the functions, secretions and excretions, and details the presenting appearances, &c. &c.—the pupil is then questioned by the professor on any point in the examination he may have omitted, and is required to give his opinion on the prognosis and diagnosis, and finally to prescribe the medicine and dietetics—all which are corrected, if necessary, by the professor, who then makes some observations on the general character of the disease, and the peculiarities of each individual case, and defines the line of treatment to be pursued. The pupil then prepares a concise written report of the case in Latin, in which tongue nearly all bedside communications between the professors and the students are conducted in Vienna;—on each subsequent visit the pupil describes the changes that have taken place in the interim, the effect of the medicines, and the state of the functions. Moreover, a tablet of tolerable dimensions is hung over each bed, specifying not only the disease, name, age, sex, and nativity of the patient, but also the medicines, diet, and regimen, the state of the pulse, the temperature, and the secretions. This judicious arrangement not only makes known to the class the precise condition of each case, but saves the patient the annoyance and injury of that continued questioning by the students, practised in other hospitals, particularly in this country."

The student cannot exercise his pleasure at what period he will attend any particular clinic, for he is not permitted to enter upon this practical branch until the fourth year of his couse, when he is fully grounded in all the elementary branches. By the course here pursued, the mind of the student is properly trained—he is systematically educated to observe disease, and, at the same time, exercise his reason and judgment, and that under competent teachers.

The chief drawback to this system is the crowded state of the clinics—three hundred students sometimes attending.

The surgery-practice is very simple, the Vienna surgeons rarely gaining a reputation at the expense of hecatombs of patients.

"Associated with the surgical clinique, is the Operators' Institute, *Das K. K. Chirurgische Operators Institut*, established by the late emperor in 1807, chiefly at the instigation of Kern.

"This is one of the most valuable institutions connected with medicine in Austria. Its object is, to educate as operators a certain number of physicians and surgeons. They are fourteen in number, have apartments in the hospital, and are supported by the state for two years, the full period required for their studies. Each of the three grades of the profession are eligible to fill these places, but they must be either doctors of medicine, doctors of surgery, or *Wundärzte*. If the former, they are required to undergo a special surgical examination, and if the latter (or patrons of surgery,) they must previously take out the degree of master surgeon (*Magister der Chirurgie.*)

"The privilege of sending pupils to this school is not confined to the Vienna University, but a certain number are sent from each of the universities yearly: the candidates are first recommended by the surgical professor, and then make a *concours* in their own university, the subject of examination being topographical or regional anatomy. The election takes place on the 8th of February, and the person elected must sign a bond, promising to remain in the Austrian states. During the first year they operate on the dead body only, and at the end of this period they undergo a public examination, when, if found proficient, they are permitted to operate on the living subject: they also receive private instruction from Professor Wattmann: and the operations of the surgical clinique, with some rare exceptions, are performed by these young men.

"Many years before the establishment of this institution, which is now connected with the university, a similar one existed in the Josephinum Academy for military surgeons. Professor Rosas instructs two of these gentlemen gratis at each of his private courses.

"At the expiration of their two years' study they receive a special diploma as *Operateurs*, and, for the most part, return to their particular provinces, where they are required to serve the state gratuitously for some time; this time is, however, seldom of long duration, for they are very soon advanced to some place of trust and emolument—indeed there are many situations under the medical government of the country that can be filled only by these persons.

"MEMBERS OF THE OPERATORS' INSTITUTE.

| | |
|---|---|
| Lower Austria | 52 |
| Upper Austria | 8 |
| Styria | 17 |
| Bohemia | 12 |
| Moravia | 3 |
| Carinthia | 1 |
| Carniola | 1 |
| Illyria and Coastland | 4 |
| The Tyrol | 3 |
| Gallicia | 6 |
| Venetian States | 15 |
| Lombardy States | 16 |
| Hungary | 7 |
| Transylvania | 5 |
| | 150 |
| Russia | 2 |
| | 152* |

"One hundred and seventy-four physicians and surgeons have been educated as *Operateurs* at this institute since its commencement, twenty-two of whom have since died; the remaining one hundred and fifty-two are distributed throughout the Austrian provinces, as in the foregoing return."

The great attraction at Vienna, however, is the Ophthalmic Clinic. Although Joseph Barth is generally claimed as the founder of the Ophthalmic School, the true founder was Nicolas Joseph Pallucci, an Italian, who, in 1745, was brought to Vienna from Florence, by Van Swieten. He depressed, but never extracted for cataract. He was the first who removed with a forceps an opaque capsule, through an opening in the cornea; an operation which Professor Jäger now frequently performs, using a hook instead of a forceps. The present Professor is Dr. Anton Edlen von Rosas, a Hungarian, who holds a daily clinic from ten to twelve o'clock. The cliinc consists of

"A male and female ward, with twenty beds, most admirably fitted up for the comfort of persons labouring under diseases of the eyes; the beds, constructed on the principle of those for fractures, and made to raise in the upper half, are furnished with curtains, the walls and fixtures painted green, and the windows so arranged as to modify the light. To prevent the chance of contagion from the indiscriminate use of the sponges and napkins, or the vessels, each ward is supplied with a small cistern, placed against the wall, about five feet from the ground, with a siphon-shaped tube attached; on turning the cock of this, a *jet* of luke-warm water plays to the height of about eight inches. To this each patient who requires ablution applies his eyes, and thus, without the fear of infection, syringes the organs in the most gentle and agreeable manner. It is an apparatus that the eye wards of every hospital should be supplied with.

"The annual average number of patients treated in this department is about one hundred and fifty, all interesting cases chosen by the professor's assistant from the general wards of the hospital."

"The business of this clinique is conducted on much the same principle as the other departments; perhaps it may be a little more methodical. The professor, the patient, and the *Ordinarius*, or attending pupil, occupy the interior of the theatre; the latter then proceeds with the examination of the case, detailing first the objective and then the subjective symptoms, the diagnosis, and the therapea. Great pains are taken by Professor Rosas to instruct his pupils in the general constitutional treatment of the patients;—an advantage which this school possesses beyond any other in Europe, except the London ophthalmic hospital in Moorefields. The treatment pursued by Dr. Rosas, in the generality of cases, is very similar to that adopted with such marked success at the latter institution, and already made known to the world by the work of my friend and preceptor, Mr. Tyrrell. Under this system, the eye-disease is in so many instances regarded but as an index to the state of general constitutional derangement, and the organ itself treated as a delicate portion of the human frame, and not a mere chemical preparation, to be altered and acted on by the different salts and compounds applied to it, as is now too much the fashion in Great Britain.

"Immediately before each operation, the attendant pupil reads to the class a Latin dissertation upon the case, its history, the objects of the operation, and the probable result. This, at least, is pure clinical instruction.

"After the ordinary duties of the clinique are ended, the professor visits those patients unable to be removed from their wards, where the most valuable information may be gleaned from his observations.

"Dr. Rosas delivers a course of systematic lectures on the diseases of the eye, on two days of the week, and his work, 'The Handbook of the Theory and Practice of Eye-surgery,' is one of the modern works upon that subject most generally read in Germany. Strangers are always admitted free of expense into this as well as the other public cliniques of the *Krankenhaus*.

"Rosas is a dexterous and steady operator. In his extraction the patient is seated on a low stool, with the head placed obliquely to the light, and resting against the breast of an assistant, who raises the upper lid, while the operator depresses the lower with the middle and forefingers in the usual manner. He makes the downward section with a knife somewhat different from that of Beer, as originally used by him, and figured in his work in 1830. This knife is much shorter in the blade than Beer's, its posterior edge (or back) is also sharp and slightly convex. Holding it between the thumb, and the index and middle fingers, the ring finger bent unto the hollow of the hand, and the little one resting on the cheek bone, he introduces the point at a right angle with the cornea, (to prevent its catching in its layers,) a little above the transverse axis of the eye, and having entered the anterior chamber, he alters the position of the instrument by depressing its handle towards the temporal fossa, and thus brings the surface of the blade on the same plane with that of the iris. Having passed it rapidly through the chamber, and made the counter punctuation, so that a full quarter of an inch of the point has passed through the inner margin of

* "Twenty-nine of these are at present, or have been professors."

the cornea, he then *draws* it slowly downwards, and slightly outwards, and so completes the section. If the case is one of double cataract, he makes the corneal section, and concludes the operation in the second eye before he extracts the lens of the first. He opens the capsule with a Langenbeck's needle, sharpened on its concave edge, and extracts the lens by gently pressing on the upper portion of the cornea with the flat of the needle.

"The object aimed at in having the back of the knife curved is, to give it shortness as well as breadth, and thus avoid pricking the side of the nose; and its posterior sharp edge is to permit of its cutting upwards as well as downwards, and thus not only pass through the cornea with greater facility, but also to enable the operator to extend the incision upwards if the original punctuation is too low. Another reason assigned by the inventor of this knife is, that its blade by being sharp at both sides, and forming in its section a compressed ellipse, permits less escape of the aqueous fluid in passing through the chamber, than the ordinary instrument.

"In this manner, Rosas operates with the most marked success; but in other hands, especially beginners, his method and instruments are open to many objections. The insertion of the knife at right angles with the cornea, is very liable to transfix the iris, and by twisting the cornea itself, renders its further insertion less smooth and easy, and its cutting back endangers both sclerotic and iris, especially in turning its lower edge outward when completing the incision; and when the iris happens to roll over the back of the knife, it cannot be pressed off with the same facility as when the posterior part is blunt; should the point of the knife get entangled in the iris, he withdraws it, and reintroduces it in another place; if the corneal opening is too small, he enlarges it with a Daviel's scissors.

"The operations of depressions and reclination are much more common in the Viennese school than in England. In this clinique these, as well as the operation for solution, are performed *per scloroticum.* In artificial pupil, Rosas generally adopts the methods of Beer and Langenbeck, but removes the portion of iris drawn through the wound.

"Professor Rosas gives a course of private instruction in operating, which, being the same as that of Jäger, will be described hereafter."

Auscultation and Pathological Anatomy are at present much attended to at Vienna. Dr. Skoda's private clinic is the best school for auscultation in the world. Skoda is an admirable auscultator, and takes great pains as a teacher. He considers all mere shades of difference in respiratory sounds as of little diagnostic value, and abolishes the names of all these embarrassing minutiæ. His divisions of the respiratory sounds are—1. Vesicular Respiration. 2. Bronchial Respiration. 3. Indeterminate Respiratory Sounds. 4. Amphoric and Metallic echoes. His divisions of the sounds of the voice are:—1. Strong Bronchophony, or the resonance of the voice, accompanied by a concussion of the ear—the voice which penetrates the stethoscope completely. 2. Weak Bronchophony, or that without, or with imperceptible, concussion of the ear. 3. The indistinct buzzing, and absence of all sound. 4. The Amphoric Echo. Instead of adopting the usual explanation of bronchophony, viz.—the dense substance of the lungs being a better conductor of sound—he explains it by consonance—namely—that the walls of the bronchial tubes being made firmer by the surrounding condensed pulmonary tissue, reflect the sound, and thus the air in the bronchial tubes vibrate in consonance with that in the larynx.

The present school of Pathological Anatomy, under the celebrated Rokitansky, has of late acquired much notoriety. The Professor, with his assistants, daily visit the dead house, and examine the majority of the bodies of those who have died of any interesting affection—from four to six bodies are opened every day—and the morbid appearances are demonstrated to the class. Rokitansky does not engage in the study or treatment of disease. Though an unrivalled pathological anatomist, he is, in reality, no pathologist, and as all his deductions are drawn from mere cadaveric appearances, his opinions on disease should always be received with great reserve. We hazard this little caution in this place, because Rokitansky's reputation is a rapidly growing one, and, as authority is every thing now-a-days in medicine, in a few years we shall not be surprised if the most astounding theories become the vogue, solely because they emanate from the fashionable Vienna Professor. His late theory on Typhus is a specimen of what we may anticipate from a man whose studies are thus exclusive. This pure objective method we cannot think calculated to advance medicine either as a science or an art. As Rokitansky is the acknowledged head of the young Viennese school, and as he is a man of great abilities, as well as a clear and attractive writer, his influence is by no means limited.

